# Finishing Fillings

**Published:** 1877-01

**Authors:** H. Judd


### FINISHING FILLINGS.

BY H. JUDD, D. D. S.

The importance of finishing fillings carefully has not generally been fully appreciated. It has been regarded by many as an ornamental, rather than a useful operation; indeed as one which enured rather to the benefit of the operator than to the patient, so that those who neglected to a great extent this part of the process of filling, solaced themselves with the idea that it did not concern their patients particularly, though it might militate somewhat against themselves in the way of reputation. Practitioners easily persuaded themselves that this was the case when considering the great amount of hard labor which would be required to cut down a huge crown filling made of hard gold, with no other way to finish but by a hand bur, which was worked by an almost infinite amount of labor—in fact hours sometimes being wearily worn away upon a single plug. I must, in all honesty, confess that the importance of fully finishing up plugs had not sufficiently impressed me so as to induce me to give my fillings all the labor and care necessary to cause them to present a really finished appearance till long after machinery had stepped in to assist us in this most irksome and seemingly endless of all our operations. The importance of a good finish is now pretty generally recognized, and especially upon proximal cavities. In crown cavities there is generally less danger of decay between the filling and the walls of the cavity than there is in proximal cavities, and the danger in the latter location is to a considerable extent proportioned to the completeness of the finish at the margin of the plug.

If the margins of a proximal plug are left unfinished, however well packed may be the gold, or with whatever care the cavity may be cleaned and the margins of the walls prepared, enough of food will be retained along the line of junction between the tooth substance and the gold to keep up, by its decomposition, an action that will finally break down the adjoining tooth structure, and we have as a consequence a cavity at the margin of the plug. The danger of this result is less on grinding surfaces, but the danger is nevertheless sufficiently great in any location to imperatively demand a careful finish, at least around the entire margins of every filling. But what should we understand by a good finish? I consider a surface which appears like the surface of a rich piece of gold plate but slightly polished, with no pits, no scratches, and with the margins of the plug clearly and smoothly defined where they join the tooth substance, to fully fill the requirements of a good finish. Hours spent in finishing to a greater degree of polish than this, are usually thrown away. With our present facilities for dressing up fillings, this finish may be easily attained upon fillings that have been properly packed. It will be observed that I particularly emphasize the words properly packed; as no amount of labor or skill spent upon a plug that has not been properly packed, can make it present a respectable appearance.

As the proper packing of the gold seems to be a "sine qua non" in attaining a fine finish, it seems proper that I should devote a little time to the consideration of this subject, even if it should seem to be beyond the limits of our proposed inquiry. I do not propose to speak of soft foil fillings, but only of those made of adhesive gold, of whatever thickness. Now, a great majority of adhesive gold fillings are packed too rapidly. If each piece of gold is not carefully consolidated before others are introduced, or if the pieces which are placed in the cavity are too large, imperfections will exist in the fillings in spite of all our efforts. I find also that the best operators use very fine points in packing the surfaces and margins of the filling, introducing also small pieces of gold, say if it be number twenty, pieces about one-fourth of an inch square; whilst on the center of large fillings, pieces of much larger size can safely be used. In packing the surfaces of fillings also benefit will be derived by carrying the packing instrument, the point of which should be square instead of being round, directly across the whole surface of the plug, then back and forth, so that the entire surface shall be covered, which is not apt to be done if the instrument is set here and there at random. I must also premise that the cavity must be prepared with an eye to finishing up the plug, or, in many cases, no amount of skill in packing, or of labor in finishing, will suffice to produce a filling which will present a really artistic appearance. If the cavity be upon the grinding surface of a molar, and the surface of the tooth is rough, cut up as it were, with ravines or ridges, or dotted over with rough projecting tubercular masses, as is often the case, these must be boldly cut way, with an emery wheel properly shaped for this purpose, and then, after the grinding surface has been sufficiently smoothed down, will it be possible to put in a filling the margins of which will be clearly defined, and the surface of which can easily be made smooth. One more suggestion, however, in regard to filling proximal fillings so that they can be properly dressed up or finished. In filling proximal cavities the most frequent error in operation arises from neglecting to keep the fillings, as they grow from the cervical margin towards the cutting edge or grinding surface, constantly contoured out as far as is necessary, so that there will be no necessity of building on to the proximal surfaces after the plug has been carried out its full length. If the fillings are not constantly kept rounded out as they progress, all attempts to round them out and supply deficiencies at the close of the operation, are apt to be futile; and rough, unsightly, or misshapen surfaces are liable to be the result. If these preliminaries which I have just noted, are carefully attended to, the finishing of fillings now, thanks to our improved instruments and implements, is comparatively an easy operation, and one which does not require much time.

The use of burs in cutting down plugs has been almost entirely superseded by the use of corundums of various shapes, from the thin disks to the almost spherical masses which are so easily made by any one, or which can be bought at the depots for a very reasonable price. If the rugosities of a molar grinding surface has been smoothed off by one of these corundums, it will be found that the filling can be cut down by the same instrument more easily than by any other, as the shape of the corundum will be such that it will exactly fit the concavity upon the grinding surface of the tooth. Whether the same corundum be used or not, the gold should be trimmed down to the margin of the cavity with the corundum wheel, when the iustrument should be changed, and a wheel of Hindostan stone, or of Scotch stone, or what is much more economical and equally as good as either of these, one made of a common fine grit sand stone, should be used for a short time all over the surface, then follow with a leather sphere with pulverized pumice, and then a little chalk or rouge on a leather base, and the finish will be found to be complete. The price of these mounted Hindostan stones is simply ridiculous. The stone is soft and soon wears out. They can easily be made by any one who has any ingenuity at all, by breaking up a Hindostan stone into pieces, drilling a hole into a piece so it can be mounted, and then it may very easily be shaped in any lathe by working it against any old corundum wheel or piece of a wheel such as is found lying around almost every cental office, Scotch stone is just about as good as Hindostan stone, and just about as easily worked; whilst a fine piece of sand stone hone makes a stone very much more valuable for this purpose than either of the others, though it is not made so easily. I am satisfied that Hindostan or Scetch stones can be made and mounted, in quantities, at an expense of not more than five cents each.

In finishing up proximal fillings the file has been to a great extent superseded by the use of the corundum disk, and the latter has also given place, to some extent, to the use of hard rubber and corundum disk, which in many places, and for certain purposes, is much superior to the corundum disks. The corundum disk, unless too thick to be available for proximal separations and for finishing proximal fillings, is entirely too brittle to answer the purpose; those which are thin nearly all the way from the center to the edge are, as a general thing, almost totally worthless; whilst those which grow thicker towards the center, although much stronger and more useful, are too thick to work in many locations where they are needed. The rubber and corundum disks may be very thin and still strong enough for use and they likewise leave a much smoother surface than the corundum, though as a general thing they cut somewhat slower than the corundum. Where there is plenty of room between the teeth, the projecting proximal filling can be cut down most satisfactorily with one of the thicker corundum disks, but the finish must be put on with a vulcanite and corundum wheel, but if the space between the teeth is not ample it will be best to do all of the cutting with one of the rubber and corundum wheels, and between the incisors, when there may be but a very narrow space, even the thinest of these will be too thick, and we shall be obliged to resort to very fine separating files for this purpose. After filing, nothing in my hands has ever proved so satisfactory as a strip of very fine sand paper to take out the scratches of the file, and this should be followed with a strip of chamois skin with pulverized pumice, and then rouge, and the finish will be sufficiently fine to satisfy the most fastidious. In lieu of sand paper I have tried many things, but none of them are equal to it, if a good sand paper is obtained. The base must be strong, the grit fine, No. o or oo, and so far as my experience goes, it is better than emery paper or any of the prepared tapes which have been in the market for many years, and which are much more expensive and much less efficient. I must not close this essay without noticing one place upon proximal fillings which, as every one knows, it is exceedingly difficult to properly finish, that is the cervical margin of the plug, and more especially when this margin is more or less overhung by the gum. A file or wheel can not reach it in many cases without seriously mangling the gum, and the only instrument which can reach the point with ease and certainty, and which will cut away rapidly the overhanging mass of gold is a very fine straight shaft, cut like a common finishing bur, but with the cuts longitudinally placed around the shaft. This must be so small that it can pass between the necks of the teeth, and with the aid of this little instrument and an engine an exceedingly troublesome operation is rendered a very simple one. If the instrument be carefully used until no overhanging patches of the plug are left, which can be easily determined by examinations with a very firm excavator, the operation may be completed at this point with a burnisher, this being the only point where a burnisher seems to be of any particular use in finishing fillings.

Amalgam fillings should always be stoned down and finished like gold ones.—*Miss. Journal.*

